# Positive rates of *Mycoplasma pneumoniae* antibodies in children before, during and after COVID-19 outbreak: an observational study in Chengdu, China from 2017 to 2024

**DOI:** 10.3389/fmicb.2025.1649615

**Published:** 2025-09-16

**Authors:** Luoman Yan, Hao Dong, Haiyan Zhang, Zixi Du, Meimei Lai, Lei Zhang

**Affiliations:** ^1^Chengdu Women's and Children's Central Hospital, School of Medicine, University of Electronic Science and Technology of China, Chengdu, China; ^2^Department of Pediatrics, The Affiliated Hospital of Southwest Medical University, Sichuan Clinical Research Center for Birth Defects, Luzhou, Sichuan, China

**Keywords:** respiratory tract infection (RTI), children, *Mycoplasma pneumoniae* (*M. pneumoniae*), epidemiology, the COVID-19 pandemic

## Abstract

**Introduction:**

*Mycoplasma pneumoniae* (*M. pneumoniae*) is a leading pathogen of pediatric pneumonia, yet its epidemiological profile in Chengdu remains understudied. This study aimed to analyze the epidemiological trends of *M. pneumoniae* antibody positive rates among children in Chengdu from 2017 to 2024, encompassing periods before, during, and after the COVID-19 pandemic, and to assess associated changes in respiratory disease patterns.

**Methods:**

We retrospectively analyzed clinical diagnoses and *M. pneumoniae* antibody test results from 222,364 children with respiratory infections treated in the emergency, outpatient, and inpatient departments of Chengdu Women and Children's Central Hospital (January 2017–December 2024). Local temperature and humidity data were concurrently collected. Epidemiological trends in *M. pneumoniae* antibody positive rates were evaluated by year, sex, age, season, and climate parameters, alongside shifts in respiratory disease composition among *M. pneumoniae*-positive children.

**Results:**

The *M. pneumoniae* antibody positive rates exhibited an overall upward trend, with three epidemic peaks (2017, 2019, and 2023–2024) and a notable decline during the pandemic. Females showed higher susceptibility than males. Outpatients aged 3–6 years and inpatients aged 0–3 years were most vulnerable pre-pandemic; however, post-pandemic, *M. pneumoniae* antibody positive rates increased with age (0–6 years). Seasonal peaks typically occurred in autumn, but during the mid-to-late pandemic, winter-autumn alternation was observed. Early-pandemic humidity positively correlated with *M. pneumoniae* antibody positive rates. Post-pandemic, asthma replaced post-infection cough as the third most common outpatient diagnosis, while inpatient diagnoses were dominated by pneumonia and severe pneumonia, the latter showing a significant rise in proportion.

**Discussion:**

Following the COVID-19 pandemic, a substantial increase of *Mycoplasma pneumoniae* (*M. pneumoniae*) antibody positive rates was observed among pediatric populations in Chengdu beginning in 2023. This study presents a descriptive analysis of serum antibody detection results, offering baseline epidemiological data to inform prevention and control strategies for *M. pneumoniae* infections among children in the Chengdu region.

## Introduction

Respiratory infections are among the most common diseases worldwide, affecting individuals of all ages. *Mycoplasma pneumoniae* (*M. pneumoniae*), a cell wall-free obligate parasitic pathogen, occupies an intermediate position between bacteria and viruses and is a significant cause of Acute Respiratory Infections (ARIs) in children. *M. pneumoniae* infections can lead to severe upper and lower respiratory tract symptoms, in excess of 10% of cases progressing to pneumonia and potentially life-threatening complications (Yang et al., 2024; Hu et al., 2023). Globally, *M. pneumoniae* outbreaks occur cyclically every 3–7 years in specific regions, with each outbreak lasting approximately 1–2 years (Yamazaki and Kenri, 2016). Following the COVID-19 outbreak in late 2019, countries implemented Non-Pharmaceutical Interventions (NPIs), including school closures, social distancing, mask mandates, enhanced hand hygiene, and restrictions on outdoor activities. These measures effectively curbed the spread of COVID-19. However, since December 2022, China began gradually relaxing NPIs, leading to significant shifts in the prevalence of *M. pneumoniae* infections (Baker et al., 2020). Since June 2023, an early peak in *M. pneumoniae* infections among children has been observed in multiple regions of China, particularly in September. The surge in cases has been accompanied by more severe clinical manifestations, with many infections involving macrolide-resistant *M. pneumoniae* strains, which are associated with worse clinical outcomes (Yan et al., 2024).

The prevalence of *M. pneumoniae* infections in children exhibits regional variations due to differences in climate, culture, and geography (Wang et al., 2025). However, limited studies have examined changes in *M. pneumoniae* infection patterns before and after the COVID-19 pandemic, and data on *M. pneumoniae* epidemiology in Chengdu remain scarce. This study retrospectively analyzed the epidemiological trends of *M. pneumoniae* antibody positive rates in children with Respiratory Tract Infections (RTIs) at Chengdu Women and Children's Central Hospital from January 2017 to December 2024. The findings provide valuable baseline data for optimizing region-specific prevention and control strategies against *M. pneumoniae* infections in children.

## Materials and methods

### Materials

The clinical diagnoses and *M. pneumoniae* antibody test results of two lakhs twenty two thousand three hundred and sixty four children with Respiratory Tract Infections (RTIs), including eighty nine thousand two hundred and sixty eight hospitalized cases, were retrospectively analyzed at the outpatient and emergency department of Chengdu Women and Children's Central Hospital from January 2017 to December 2024. The cohort also comprised one lakh thirty three thousand hundred and seventy eight outpatients; And with one lakh twenty one thousand four hundred and eighty eight males and one lakh eight hundred and seventy six females with a sex ratio of 1.2:1. Age groups included infants and young children (0 ≤ age ≤ 3 years old), preschoolers (3 < age ≤ 6 years old) and school-age children (6 < age ≤ 14 years old). Climate data (temperature and humidity, 2017-2024) were sourced from the Sichuan Meteorological Bureau's official records. The study period was divided into pre-pandemic (2017-2019), pandemic (2020-2022), and post-pandemic (2023-2024).

### Inclusion criteria

(1) Clinical diagnosis of respiratory tract infection according to WHO criteria; (2) Availability of *M. pneumoniae* antibody testing; (3) Complete *M. pneumoniae* antibody test results and demographic data; (4) Age 0-14 years; (5) Presence of characteristic clinical manifestations (e.g., paroxysmal cough, persistent fever ≥38 °C, or radiologically confirmed pulmonary abnormalities); (6) No record of *M. pneumoniae* antibody positivity in the prior 6 months; (7) Single serum *M. pneumoniae* antibody titer ≥1:160 (PA method), indicating a positive result.

### Statistical analysis

Data were analyzed by age, gender, year, month, and climate. Categorical variables were expressed as percentages, and the Chi-square test (χ^2^) was used for comparisons. Bivariate correlation analysis was performed to assess relationships. Statistical significance was set at *P* < 0.05. All analyses were conducted using IBM SPSS 27.0 software.

## Results

### Analysis of gender and age differences in children with *M. pneumoniae* antibody positive rates before, during and after the pandemic

**Figure 1:**
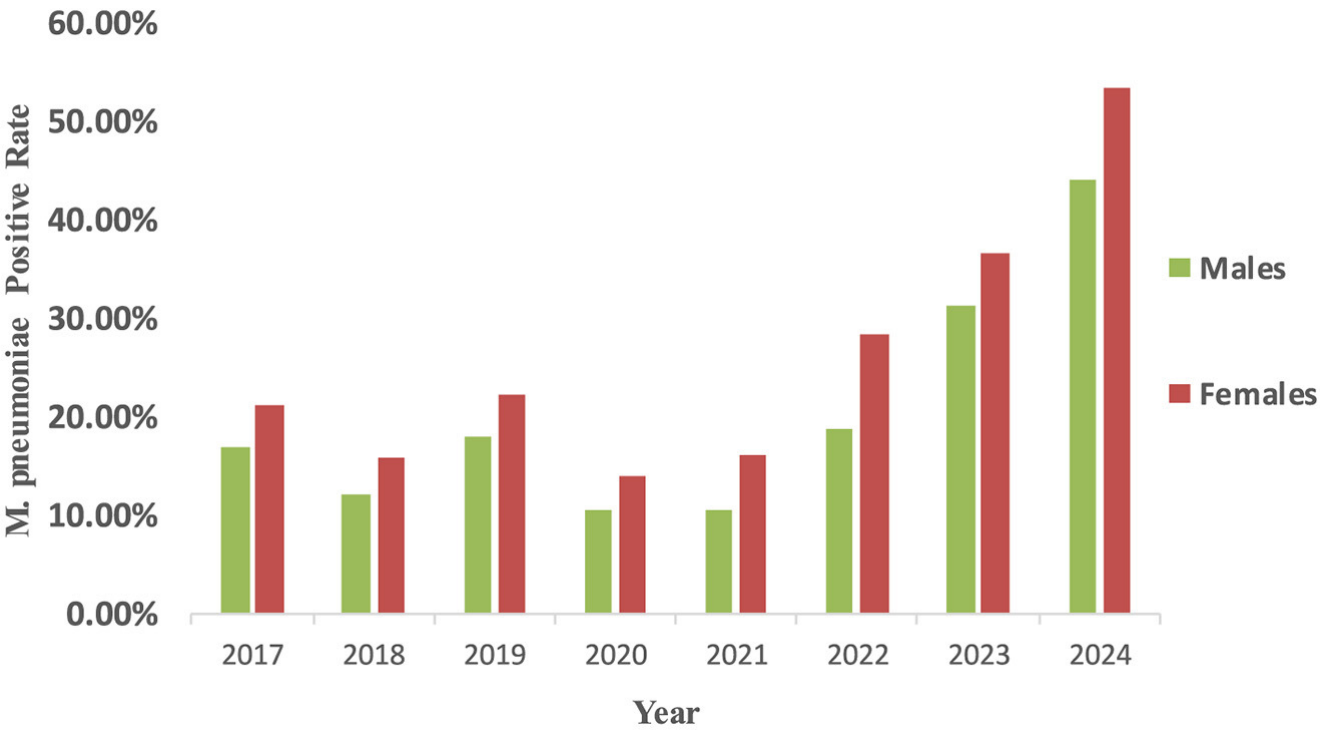
Epidemiological changes of *M. pneumoniae* antibody positive rates according to patient gender. *M. pneumoniae* positive rate were computed as the proportion of *M. pneumoniae* antibody positive individuals among all tested individuals of each gender. The detection of serum antibodies merely reflects prior exposure to *M. pneumoniae* and does not constitute definitive evidence of acute infection.

**Figure 2:**
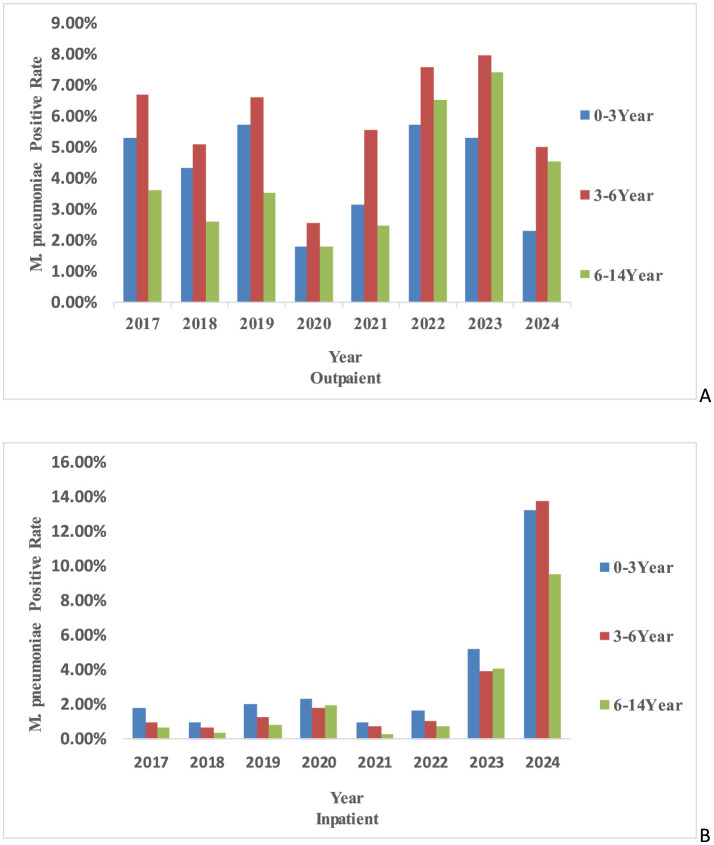
The age-specific trends of *M. pneumoniae* antibody positive rates. **(A)** The age-specific trends in outpatient *M. pneumoniae* antibody positive rates in different years. **(B)** Age-specific trends in inpatient *M. pneumoniae* antibody positive rates in different years.

From 2017 to 2024, the *M. pneumonia* antibody positive rates was significantly higher in female children than in males (χ^2^ = 135, *P* < 0.001) (Figure 1). Age-specific differences in *M. pneumoniae* antibody positive rates were also observed during this period (χ^2^ = 693, *P* < 0.001). Age groups included one lakh twenty one thousand three hundred and ninety eight infants and young children, sixty six thousand hundred and seventy three preschoolers and thirty four thousand eight hundred and ninety three school-age children. Notably, the antibody positive rates increased with age (0-6 years) in the later stages of the pandemic. Pre-pandemic, preschool-aged children (3–6 years) were the most affected group, followed by infants and young children (0–3 years) and school-age children (6–14 years). Post-pandemic, the antibody positive rates among school-age children surpassed that of infants and young children, becoming the second most affected group after preschoolers (Figure 2A). Among hospitalized children, preschoolers had higher *M. pneumoniae* antibody positive rates than infants and young children in 2024 (Figure 2B).

### Analysis of differences in *M. pneumoniae* antibody positive rates across years before, during and after the pandemic

**Figure 3:**
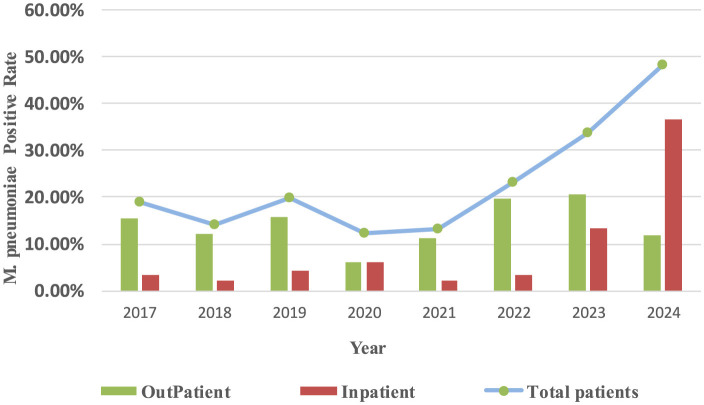
Yearly trends in *M. pneumoniae* antibody positive rates in different hospitalization methods and total positive rates.

From 2017 to 2024, the *M. pneumoniae*-positive rates in outpatients were 14.47%, 12.69%, and 16.18% during the pre-pandemic, pandemic, and post-pandemic periods, respectively. For inpatients, the corresponding rates were 3.24%, 3.82%, and 24.97%. Pre-pandemic, *M. pneumoniae* antibody positive rates exhibited a biennial increase, with epidemic peaks occurring in 2017, 2019 and 2023–2024. Post-pandemic, the *M. pneumoniae*-positive rate continued to rise (Figure 3).

### Analysis of monthly *M. pneumoniae* antibody positive rates and environmental temperature and humidity before, during and after the pandemic

**Figure 4:**
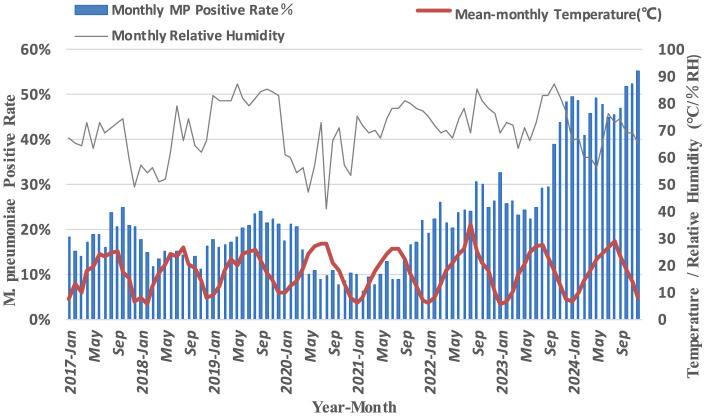
Monthly *M. pneumoniae* antibody positive rate and environmental temperature and humidity.

Significant monthly variations in *M. pneumoniae* antibody positive rates were observed from 2017 to 2024 (*P* < 0.001). Pre-pandemic, antibody positive rates were consistently higher from August to December each year. Post-pandemic, the peak duration of *M. pneumoniae* antibody positive rates extended, though no significant correlation was found with monthly temperature changes (*P* > 0.05) (Figure 4). A positive correlation was identified between *M. pneumoniae*-positive rates and environmental humidity before the pandemic (*P* < 0.01). However, this correlation was not observed in the post-pandemic period (*P* > 0.05) (Table 1).

**Table 1:** Correlation analysis between monthly *M. pneumoniae* antibody positive rates and environmental temperature and humidity.

**Time**	**Mean-monthly temperature**	**Monthly relative humidity**
2017/1-2019/12	0.175	0.463^**^
2020/1-2022/12	0.034	0.358^*^
2023/1-2024/12	−0.183	−0.226

^**^P < 0.01, ^*^P < 0.05.

### Comparison of the proportion of respiratory diseases in children with *M. pneumoniae* antibody positive

**Figure 5:**
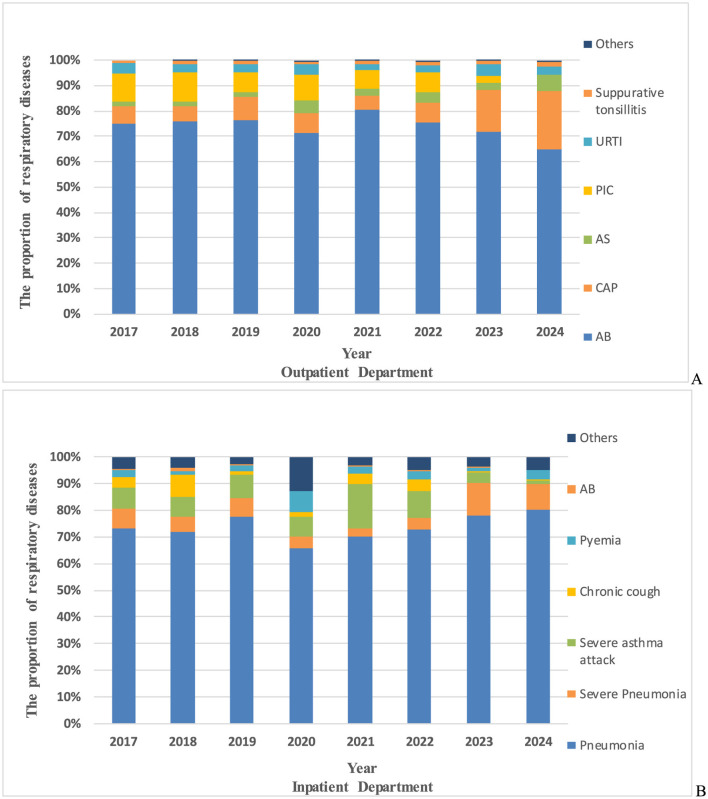
The proportion of respiratory diseases in *M. pneumoniae* antibody positive. **(A)** The proportion of respiratory diseases in *M. pneumoniae* antibody positive in outpatient department. **(B)** The proportion of respiratory diseases in *M. pneumoniae* antibody positive in inpatient department. AB, acute bronchitis; PIC, post-infectious cough; CAP, community acquired pneumonia; URTI, upper respiratory tract infection; AS, asthma; others, respiratory diseases other than these.

Significant differences were observed in the spectrum of respiratory diseases associated with *M. pneumoniae* antibody positive among outpatients and inpatients from 2017 to 2024 (χ^2^ = 2,447, *P* < 0.001). Among outpatients, the top three diagnoses for *M. pneumoniae* antibody positive before and during the pandemic were Acute Bronchitis (AB), pneumonia, and Post-Infectious Cough (PIC). However, post-pandemic, asthma replaced PIC as the third most common diagnosis, with AB and pneumonia remaining the top two (Figure 5A). For hospitalized patients, the leading *M. pneumoniae*-related diagnoses before the pandemic were pneumonia, severe pneumonia, and severe asthma attacks. During the pandemic, the ranking shifted slightly, with pneumonia remaining the most common, followed by severe asthma attacks and severe pneumonia. Post-pandemic, the primary diagnoses among hospitalized *M. pneumoniae*-positive children were pneumonia and severe pneumonia, with a notable increase in the proportion of severe pneumonia cases (Figure 5B).

## Discussion

This study identified significant gender differences in *M. pneumoniae* antibody positive rates, with female children exhibiting higher rates than males, consistent with previous findings (Mao et al., 2025; Ai et al., 2024; Zhang et al., 2021). This suggests that females may be more susceptible to be positive for *M. pneumoniae* antibodies, potentially due to differences in activity and social behaviors. Female children may engage more frequently in indoor group activities, which could facilitate *M. pneumoniae* transmission in enclosed environments. While *M. pneumoniae* primarily affects children, it can infect individuals of all ages, including infants and the elderly. Previous studies indicate that *M. pneumonia*-positive predominantly occur in children over 5 years old (Zhang et al., 2025). During 2017–2024, preschool-aged children were the most susceptible outpatient group to *M. pneumoniae* antibody positive in Chengdu. Pre-pandemic, infants had the second-highest antibody positive rates after preschoolers. However, during and after the pandemic, *M. pneumoniae* antibody positive rates increased with age (0-6 years), and by 2022–2024, school-age children exhibited higher antibody positive rates than infants, aligning with studies from Wuhan, Henan, and Chengdu (Mao et al., 2025; Zhang et al., 2021; Li et al., 2023). Among hospitalized children, infants were the most susceptible group, with preschoolers showing higher antibody positive rates than school-age children, consistent with prior research. Notably, in 2020 and 2023, school-age children had higher *M. pneumoniae* antibody positive rates than preschoolers, possibly due to their broader and more active social interactions following the relaxation of NPIs (Miao et al., 2025). Additionally, the rise in macrolide-resistant *M. pneumoniae* strains, which peaked in Taiwan in 2020 (Wu et al., 2024), may have contributed to increased hospitalization rates among school-age children due to treatment challenges. This trend is further substantiated by recent findings demonstrating a significant escalation in the mutation rate of macrolide resistance loci in *M. pneumoniae* across mainland China, with 2023 data showing a marked increase compared to the 2019 to 2022 surveillance period (Xu et al., 2024). Geographical and pandemic-related factors also influenced age-specific susceptibility. For instance, in Anhui, *M. pneumoniae*-positive cases were primarily observed in children aged 7–10 years pre-pandemic, shifting to 6–14 years post-pandemic, with low antibody positive rates among infants (Chen et al., 2024), consistent with patterns in France and Denmark (Edouard et al., 2024; Nordholm et al., 2024). Conversely, in Shanghai, post-pandemic *M. pneumoniae*-positive rates increased among infants, suggesting a trend toward younger susceptible age groups (Zhu et al., 2024). These variations may be attributed to regional differences in childcare practices, school environments, and climatic conditions. Immature immune systems, crowded indoor settings, and close contact among children likely exacerbate *M. pneumoniae* transmission. Furthermore, air quality and environmental factors, such as PM 2.5 and PM 10 levels, have been linked to *M. pneumoniae*-positive, as these pollutants can carry respiratory pathogens (Gu et al., 2024).

This study also revealed that *M. pneumoniae* antibody positive rates in Chengdu peaked in 2017, 2019 and 2023–2024, consistent with previous reports (Wang et al., 2024). Pre-pandemic, *M. pneumoniae* antibody positive rates exhibited a biennial increase, but during the pandemic, outpatient rates declined significantly, with a slight rebound in 2022. Our study revealed a significant decline in *M. pneumoniae* antibody positive rates during 2020. This epidemiological pattern may be attributed to the comprehensive infection control measures and stringent case management policies enacted during the pandemic period. In China, public health strategies prioritized the treatment of severe COVID-19 cases while enforcing standardized isolation protocols for mild infections. A research reveal that during the pandemic, a proportion of COVID-19 cases were managed outside hospital settings. Furthermore, surveillance limitations were evident, as a number of symptomatic individuals remained untested, potentially resulting in underreporting of positve rates (Wu et al., 2025). This observation may similarly apply to *M. pneumoniae* antibody positive rates. And the overall decline in *M. pneumoniae* antibody positive during the pandemic also has been attributed to NPIs, such as personal protective measures and social distancing. A study has shown the *M. pneumoniae* positive rate during the NPI phase was significantly lower than that during the non-NPI phase, our results align well with these observations (Jiang et al., 2024). Post-pandemic, both outpatient and inpatient *M. pneumonia*e antibody positive rates rose sharply, with inpatient rates surpassing outpatient rates. This trend may reflect increased macrolide resistance and the “immunity debt” hypothesis. Supporting this notion, recent surveillance data from Henan and Beijing, China, demonstrate a marked increase in the proportion of macrolide-resistant *M. pneumoniae* isolates since 2023 compared to pre-pandemic levels (Qi et al., 2025; Jia et al., 2024). The latter hypothesis posits that prolonged implementation of NPIs during the COVID-19 pandemic substantially reduced population-wide pathogen exposure, consequently impairing immune surveillance and increasing susceptibility to *M. pneumoniae* positive. This hypothesis has gained empirical support through observed epidemiological patterns of various respiratory pathogens, most notably Respiratory Syncytial Virus (RSV), during their post-pandemic resurgence periods (Munro and House, 2024), thereby corroborating our current findings. Seasonal patterns of *M. pneumoniae* antibody positive also shifted during the study period. Pre-pandemic, antibody positive rates peaked in autumn (August–October), consistent with findings from Beijing (Wang et al., 2022). However, during and after the pandemic, seasonal peaks alternated between winter and autumn, extending into the following spring. Notably, in 2022, the peak shifted to spring and summer, likely due to pandemic-related disruptions in typical seasonal transmission patterns (Edouard et al., 2024). Regional variations in seasonal trends were also observed. For example, in Shanghai and Wuhan, *M. pneumoniae*-positive rates peaked in autumn and winter 2023, persisting into spring 2024 (Edouard et al., 2024; Mao et al., 2025), while in Northeast China and Inner Mongolia, peaks shifted from winter and spring to summer and autumn (Wang et al., 2024). These changes underscore the impact of COVID-19 on *M. pneumoniae* seasonality (Ai et al., 2024). Early in the pandemic, environmental humidity positively correlated with *M. pneumoniae* positive rates (Wagatsuma, 2024), a study by Japanese scientists has identified a significant positive association between the incidence of *M. pneumoniae* positive rates and weekly average temperature and humidity levels (Onozuka et al., 2009). But our finding has shown that this relationship weakened post-pandemic, possibly due to increased parental awareness and interventions such as air purifiers and cleaning behaviors (Liu et al., 2025).

The spectrum of respiratory diseases among *M. pneumoniae*-positive children also evolved post-pandemic. In outpatient settings, asthma replaced post-infectious cough (PIC) as the third most common diagnosis. This shift may reflect reduced exposure to environmental pathogens during the pandemic, leading to immune dysregulation and heightened allergic responses upon re-exposure (Sampath et al., 2023). Current research substantiates that children with acute asthma had significantly higher seropositivity for anti- M. pneumoniae IgM antibodies than children with stable asthma (Liu et al., 2021) and asthmatic children with *M. pneumoniae*-positive exhibit elevated serum levels of both IL-4 and IFN-γ (Xiao and Hou, 2025). Notably, IL-4 has been clinically established as a key mediator associated with asthma susceptibility and disease pathogenesis (Jin and Zheng, 2022). Among hospitalized children, severe pneumonia replaced asthma as the second most common diagnosis, likely due to the rise in macrolide-resistant *M. pneumoniae* strains and the “immunity debt” phenomenon, which predisposed children to more severe respiratory complications (Miao et al., 2025; Qiu et al., 2024). A study has shown that the clinical manifestations of drug-resistant *M. pneumoniae* pneumonia are more severe and the hospital stay is longer (Jiang et al., 2023). Similar trends were observed in Zhejiang, Guangzhou, Northeast China, and Inner Mongolia, where pneumonia and severe pneumonia predominated among hospitalized *M. pneumoniae*-positive children post-pandemic (Wang et al., 2024; Qiu et al., 2024). These findings highlight the need for heightened vigilance and early intervention in managing *M. pneumoniae*-related respiratory diseases, particularly pneumonia, severe pneumonia, and asthma, in the post-pandemic era. This study has several limitations. First, this observational study utilized single-point serum *M. pneumoniae* antibody detection as a surveillance tool, rather than employing laboratory-confirmed acute infection criteria through paired serological testing or PCR confirmation, which cannot distinguish between acute infection and persistent antibodies from previous exposures. This approach may consequently overestimate the true prevalence of acute *M. pneumoniae* infections in the pediatric population. Second, data were limited to Chengdu Women and Children's Central Hospital, excluding *M. pneumoniae* resistance gene profiles and cases from other regional hospitals. The relatively narrow geographic scope and sample size may limit the generalizability of the findings. Given the methodological constraints of single-timepoint serology in distinguishing acute versus historical *M. pneumoniae* infections, our reported positivity rates represent the combined effects of population seroprevalence and new infections. Future investigations should integrate pathogen genomic surveillance with longitudinal serological monitoring to more precisely characterize the true epidemic dynamics of *M. pneumoniae*.

## Conclusion

In summary, this study presents a comprehensive retrospective analysis the epidemiological trends of *M. pneumoniae* antibody positive rates in Chengdu spanning from 2017 to 2024, with particular emphasis on comparative assessments before, during, and after the COVID-19 outbreak. The findings provide valuable baseline data for local healthcare practitioners in enhancing early diagnosis and preventive strategies against *M. pneumoniae* infections. Given the distinct epidemiological patterns of *M. pneumonia* positive rates, region-specific and demographic-targeted prevention strategies should be implemented for children. Our analysis of the *M. pneumoniae* antibody positive disease spectrum reveals dynamic epidemiological shifts, particularly a marked increase in asthma and severe pneumonia cases in the post-pandemic period. These findings carry substantial clinical implications, underscoring the need to adapt current diagnostic and therapeutic strategies for *M. pneumoniae* antibody positive. Further research is warranted to elucidate the underlying mechanisms, including potential alterations in host immune responses or pathogen evolution. Furthermore, our study highlights the critical need for heightened vigilance during the post-holiday period and initial school reopening phases. We recommend implementing enhanced protective measures and prompt diagnostic testing upon symptom onset to facilitate early intervention and effective disease management.

## Data Availability

The raw data supporting the conclusions of this article will be made available by the authors, without undue reservation.
